# Acceptability of screening for mental health difficulties in primary schools: a survey of UK parents

**DOI:** 10.1186/s12889-018-6279-7

**Published:** 2018-12-22

**Authors:** Emma Soneson, Jasmine Childs-Fegredo, Joanna K. Anderson, Jan Stochl, Mina Fazel, Tamsin Ford, Ayla Humphrey, Peter B. Jones, Emma Howarth

**Affiliations:** 1 Department of Psychiatry, University of Cambridge, Herchel Smith Building, Cambridge, CB2 0SZ UK; 2 NIHR CLAHRC East of England, University of Cambridge, Institute of Public Health, Douglas House, 18 Trumpington Road, Cambridge, CB2 8AH UK; 3 Department of Psychiatry, University of Oxford, Warneford Hospital, Oxford, OX3 7JX UK; 4 University of Exeter Medical School, South Cloisters, St Luke’s Campus, Exeter, EX1 2LU UK

**Keywords:** Mental health, Schools, Identification, Screening, Health checks, Acceptability, Feasibility

## Abstract

**Background:**

Many children and young people experiencing mental health difficulties (MHD) do not access care, often due to inadequate identification. Schools have a unique potential to improve early identification; however, evidence is limited regarding the acceptability of school-based identification programmes. This study aimed to examine parents’ beliefs about the acceptability of school-wide MHD screening in primary schools.

**Methods:**

We collaborated with experts in school-based mental health to develop a questionnaire to measure parental attitudes toward school-wide MHD screening. The questionnaire contained 13 items relating to acceptability; three open-text boxes for comments on harms, benefits, and screening in general; and four questions that captured demographic information. Parents of children attending four primary schools in Cambridgeshire and Norfolk completed the questionnaire. We calculated counts, percentages, and means for each statement, and analysed responses to open-ended questions using content analysis.

**Results:**

Two hundred ninety parents returned the questionnaire across the four schools (61% response rate). In the 260 questionnaires analysed, a total of 254 parents (98%) believed that it is important to identify MHD early in life, and 251 (97%) believed that schools have an important role in promoting pupils’ emotional health. The majority of parents (*N* = 213; 82%) thought that screening would be helpful, although 34 parents (13%) thought that screening would be harmful. Perceived harms of screening included inaccurate identification, stigmatisation, and low availability of follow-up care. There was no clear consensus regarding how to obtain consent or provide feedback of screening results. There were no significant differences in responses according to ethnicity, gender, age, or school.

**Conclusions:**

Results suggest that most parents within the socio-demographic context of our study will accept MHD screening within primary schools, and that school-based screening is viable from the perspective of parents. The comments provided about potential harms as well as suggestions for programme delivery are relevant to inform the development and evaluation of acceptable and sustainable school-based identification models. Implementation and scale-up of such programmes will require further understanding of the perspectives of mental health professionals, school staff, and the general public as well as further evaluation against the established standards for identification programmes.

**Electronic supplementary material:**

The online version of this article (10.1186/s12889-018-6279-7) contains supplementary material, which is available to authorized users.

## Background

The high burden of mental health difficulties (MHD) in children and young people (CYP) has been recognised as a key challenge in public health [1]. In the United Kingdom (UK) it is estimated that one in ten CYP aged 5–16 years has a clinically relevant mental disorder, and that many more experience sub-clinical symptoms that also cause significant distress and functional impairment for CYP and their families [2]. Children and young people who experience MHD can face a number of immediate and short-term consequences, including impacts on educational outcomes such as poor achievement, truancy, and increased school drop-out as well as peer difficulties and low self-esteem [2–6]. If untreated, a significant portion of MHD are likely to persist [7] and for some contribute to poor physical health, increased mortality, alcohol dependence, criminal behaviour, unemployment, and suicide attempts [3, 5, 8–10].

Mental health prevention and early intervention programmes, particularly those delivered in school settings and those that use a tiered approach of both universal and indicated prevention strategies [11, 12], show promise for reducing negative mental health outcomes [13–20] (see Fazel et al. 2014 [13] for an overview of school-based interventions). Yet, despite the availability of evidence for such programmes, many CYP experiencing symptoms of mental ill health do not access mental health services or other avenues of care [21]. There are several acknowledged barriers to accessing treatment, including availability of care, lack of information about services, inflexible services, waiting times, complex administrative procedures, treatment costs, and users’ expectations of providers’ attitudes [22]. Another significant contributor to this unmet need is inadequate identification of MHD [23, 24]; only 0.6 to 16% of CYP experiencing MHD are identified by frontline non-mental health professionals such as teachers and primary health care providers [23, 25].

### Role of schools in identifying children at risk of poor mental health outcomes

Schools can play a unique role in the early identification of MHD, particularly as they are the setting in which most mental health related service contacts currently occur [24, 26–29]. From a practical standpoint, schools reach the vast majority of CYP in the UK [23], including CYP from marginalised populations, who experience MHD at higher rates than their peers and access care less readily [2]. Furthermore, the large number of contact hours means that teachers and other school staff are well placed to notice changes in behaviour and demeanour that may signal that a student is experiencing MHD [30]. Finally, the majority of all lifetime mental disorders will begin during the schooling years [31], emphasising the opportunity that schools offer for early identification and intervention.

The UK government has emphasised the role of schools in identifying CYP with MHD and linking them with care and support [32]. The National Institute for Health and Care Excellence and the Commission on Children and Young People’s Mental Health expect teachers to be able to identify and assess early signs of MHD in their pupils [33, 34]. The UK Education and Health Committees further note that schools have a unique insight into pupils and are well placed to recognise difficulties, including those not identified at home or to complement what is observed at home [35]. However, despite these expectations, teachers often feel ill-equipped to recognise signs of MHD [12, 36], especially as mental health is poorly covered in most teacher training curricula.

### School-wide screening for MHD

School-wide screening programmes have received significant attention for their potential to identify MHD in CYP [13, 24, 27, 37]. These programmes feature systematic identification of pupils at-risk for poor mental health outcomes, as identified by the presence of symptomatology or functional impairment. School-wide screening programmes show some promise in terms of enhancing identification and linking CYP with appropriate support [38].

In addition to effectiveness, acceptability must be a key consideration in the development and evaluation of school-based identification programmes. The World Health Organization identifies acceptability as a cornerstone of any successful screening programme [39]. Similarly, the UK National Screening Committee cites acceptability as one of the key criteria that must be met before a screening programme can be implemented [40]. Acceptability is particularly important in the context of screening for MHD due to associated stigma [27, 37] and significant difficulties surrounding schools’ communication and cooperation with mental health services [41].

### Current study

The current study reports results of a cross-sectional survey of parental attitudes towards school-wide screening for emotional and behavioural difficulties in four UK primary schools. The survey is part of the DEAL (Developing Early Identification and Access in Learning Environments) study, which aims to develop an evidence-based prototype school-based programme for the identification of and response to MHD in primary school children. DEAL focuses on primary schools for epidemiological, policy, and pragmatic reasons. First, many lifetime mental disorders begin during the primary school years (for example, anxiety and impulse control disorders each have a median age of onset of 11 years [31]). Second, national UK guidelines have set forth expectations for the identification of MHD in primary schools [33]. Finally, from a practical standpoint, there are often stronger links between schools and parents of primary school aged children compared with those of secondary school aged children [42].

In order to inform programme design, the survey sought to (1) assess whether school-wide MHD screening was acceptable ‘in principle’ to parents of primary school children, (2) determine some of the preferred characteristics of an acceptable screening programme, and (3) identify anticipated benefits and harms of screening. The results from this survey, in combination with other components of the DEAL study (i.e. evidence reviews and in-depth parent and school staff interviews surrounding the strengths and limitations of multiple identification methods) will inform the design of an identification model. Furthermore, the findings from the survey are among the first published from UK data on the acceptability of screening in primary school settings, and can inform the debate on the practical role that schools can potentially play in delivering the UK public mental health response.

## Methods

### Participants

The research team recruited four schools to participate in the study. Given limited resources, we decided to focus on schools in socially deprived areas due to the higher burden of MHD in children of lower socioeconomic status [43]. To reduce heterogeneity of deprivation characteristics, we divided schools in the counties of Norfolk and Cambridgeshire into tertiles depending on the deprivation of their catchment areas, as ranked by the Index of Multiple Deprivation [44]. We aimed to select four schools serving communities from the top third of social deprivation and with above average uptake of free school meals (14.3% for England in 2016) [44]. Three of the selected schools met both criteria, and the fourth had a higher than average uptake of free school meals but was situated in a less deprived area (see Table 1 for school profiles).

**Table 1:** Characteristics of schools included in the study

	School A	School B	School C	School D
Community Indicator				
County	Norfolk	Norfolk	Cambridgeshire	Cambridgeshire
Rural vs urban	Urban	Urban	Urban	Rural
School type	Community school	Community infants and nursery school	Community school	Community school
Area IMD^a^	1	3	7	3
% White British	83.1	88.9	87.6	95.1
% English as a first language	86.0	89.8	88.9	94.4
School Indicator				
Age range	4–10	3–7	4–10	4–10
Funding	State funded	State funded	State funded	State funded
Pupils (rounded)	> 300	> 200	> 600	< 100
% Free school lunch^b^	31.0	37.9	18.3	27.0
% SEND^c^	21.0	19.0	9.0	8.0
% SEMH^d^	5.0	9.0	2.0	5.0

^a.^IMD: Index of Multiple Deprivation

^b.^Free/reduced cost school lunch (see https://www.gov.uk/apply-free-school-meals for how parents can qualify for free/reduced cost school meals)

^c.^SEND: special educational needs and disability (defined as having a learning difficulty or disability requiring special educational provision)

^d.^SEMH: social, emotional, and mental health needs

### Instrumentation

Due to the lack of an existing measure of parental attitudes toward MHD screening, we collaborated with experts in school-based mental health to develop a questionnaire to measure the acceptability of screening. We used findings from a review of the literature and known uncertainties about the acceptability of the screening process to develop the questionnaire. Questionnaire items aimed to understand parents’ opinions regarding early identification of MHD, schools’ role in MHD identification, anticipated harms and benefits of screening, and key components of acceptable screening programmes. Patient and Public Involvement (PPI) groups, an occupational psychologist, heads of participating schools, and other school staff reviewed the questionnaire. PPI members suggested simplifying wording, shortening the questionnaire, highlighting information about the participation incentive, and excluding two questions on family life and parent educational qualifications. All stakeholders approved the final version of the questionnaire. We did not compute a generic reliability estimate for the questionnaire given that it had no overall score or underlying construct.

The first page of the anonymous questionnaire (Additional file 1: Appendix C) explained the purpose of the questionnaire, defined emotional health and emotional health difficulties, explained what was meant by ‘screening’ and ‘emotional health checks’ (used synonymously throughout the questionnaire), and gave instructions for completion. The questionnaire explained screening as ‘the collection of information about the emotional health and wellbeing of all children in a school’ in order to ‘detect people with early signs of a problem so that they can be offered help at the earliest opportunity.’ As this study aimed to understand parents’ attitudes toward the general concept of screening rather than toward a specific screening programme, we purposely did not give detailed information on how data would be collected, who would fill out screening questionnaires, etc., and instead included questions to determine parents’ preferences regarding these types of programme components.

The questionnaire contained 20 questions. Thirteen statements relating to the acceptability of screening featured a 5-point Likert-type scale (*where 1 = strongly disagree and 5 = strongly agree*) and included the additional answers of *don’t know* and *prefer not to say*. The questions were heterogeneous, so we did not calculate an overall score. Open-text boxes where parents could provide additional comments followed two questions about the perceived harms and benefits of screening. In addition, a final open-text box invited parents to share thoughts or comments about screening that were not addressed by other questions. Four final questions asked parents for their age, gender, ethnicity, and the number of children they had in each school year.

### Procedures

We collected questionnaires between 17 July 2017 and 12 January 2018. Schools communicated initial information about the DEAL study to parents via mailings, letters in book bags, video clips, in-person communications, Facebook posts, and information presented on school websites, and sent regular reminders to complete the questionnaire. Furthermore, members of the research team attended schools’ parents’ evenings to encourage participation and answer any questions about the study. Questionnaire packs were distributed to parents through various routes, including in-person at school parents' evenings and via children’s book bags. Each pack contained an invitation letter, a participant information sheet, and a questionnaire (including an explanation of content and instructions for completion) (see Additional file 2: Appendix A, Additional file 3: Appendix B, Additional file 1: Appendix C). The participant information sheet (Additional file 3: Appendix B) included detailed information on the study and participation. We indicated on this sheet that participation was voluntary and anonymous, explained the advantages and disadvantages of participating, and described how data would be used. We further explained that parents could give contact details to participate in an interview, and provided information about confidentiality, data storage, and advantages/disadvantages of participating in the interview. We provided contact details for further questions or concerns and ascertained parents’ informed consent by a ticked box on the online version of the questionnaire, or the receipt of a completed paper version.

Questionnaires were also available online through Qualtrics’ online survey platform (www.qualtrics.com). Heads of schools received school-specific links for the electronic version of the questionnaire to distribute via their preferred communication routes (e.g. email, text message, Facebook). To minimise response burden, we instructed parents to fill out only one survey (either hard copy *or* electronic) per family, regardless of the number of children enrolled in the school. Parents could return completed questionnaires via their child or mail them to the research team using a free post envelope. Parents could also indicate they were not interested in participating in the study by not returning a questionnaire or by returning a blank questionnaire.

Parents from each school who participated in the study were entered into a prize draw for £50 of shopping vouchers. Hard copy questionnaires contained a raffle ticket, and online versions contained randomly-generated raffle numbers that could be printed out or written down. Parents could also enter into the prize draw without completing the questionnaire by returning a blank copy. We also awarded a £50 voucher to the school with the highest response rate.

### Analysis

#### Quantitative analysis

We used *R* for all statistical analyses [45]. We calculated overall and per-school response rates using the number of families in the school as the denominator. For all questionnaires that had at least 50% complete data, we calculated means (where 1 *= strongly disagree* and 5 = *strongly agree*, i.e. higher mean scores indicate greater agreement), counts, and percentages for responses to each of the 13 statements on screening acceptability (Additional file 4: Table S1). We used Fisher’s exact tests to determine whether responses differed by ethnicity (White British vs. other), gender, and school, and Holm corrections to adjust for multiple testing. We used polyserial correlations to determine associations with age.

#### Qualitative analysis

To analyse the open-ended comments, we used conventional content analysis, as described by Hsieh and Shannon [46]. We aimed to examine parents’ views regarding the perceived benefits and harms of screening. Since the literature in this area is not well-defined, we used an inductive approach whereby we did not have pre-defined coding frameworks [46, 47]. One researcher (ES) read the comments verbatim and created and assigned codes to each comment (multiple codes were allowed for each response). The researcher examined codes for similarity and grouped them into sub-themes, which were then grouped into broader themes to create meaningful clusters [46, 47]. To prepare for reporting the findings, the researcher identified exemplars for each code and category from the data. A second researcher (JC-F) independently assessed a subset of 15% of the comments in order to improve the rigour of the qualitative research process [48]. The two researchers discussed differences and established consensus by refining subthemes. As recommended by O’Cathain and Thomas (2004), counts for each sub-theme were generated [49].

## Results

### Parent characteristics

In total, 290 parents across the four schools responded to the questionnaire, representing an overall response rate of 61% (Table 2). Of these 290 parents, 128 (44%) responded to the open-ended question on potential benefits of screening (Statement 4), 83 (29%) responded to the question on potential harms (Statement 5), and 62 (21%) provided general comments on the DEAL study. In total, 225 (78%) questionnaires were submitted in hard copy and 65 (22%) were submitted online. Six questionnaires were returned blank and 24 were not analysed due to incomplete data (< 50% complete). We analysed 260 questionnaires in total.

**Table 2:** Characteristics of questionnaire respondents

Age *Mean (SD) (N = 242)*	37.5 (7.4)
Gender *N (%) (N = 249)*	
Female	200 (80.3)
Male	47 (18.9)
Transgender	1 (0.4)
Prefer not to say	1 (0.4)
Ethnicity *N (%) (N = 253)*	
White - English/Welsh/Scottish/Northern Irish/British	233 (92.1)
White - Any other White background	8 (3.2)
Mixed/Multiple ethnic groups -White and Asian	1 (0.4)
Mixed/Multiple ethnic groups - Any other Mixed/Multiple ethnic background	1 (0.4)
Asian/Asian British - Indian	1 (0.4)
Black/African/Caribbean/Black British - African	4 (1.6)
Black/African/Caribbean/Black British - Caribbean	1 (0.4)
Prefer not to say	1 (0.4)
Parents with children in each school year *N (%)*^a^	
Reception	43 (16.5)
Year 1	39 (15)
Year 2	53 (20.4)
Year 3	45 (17.3)
Year 4	38 (14.6)
Year 5	49 (18.9)
Year 6	71 (27.3)
Responses per school *N (% total responses)*^b^	
A	119 (45.8)
B	21 (8.1)
C	88 (33.9)
D	32 (12.3)

^a^*N* > 260 because some parents have more than one child enrolled

^b^Schools are identified by letter to maintain confidentiality

### Questionnaire responses

**Fig. 1:**
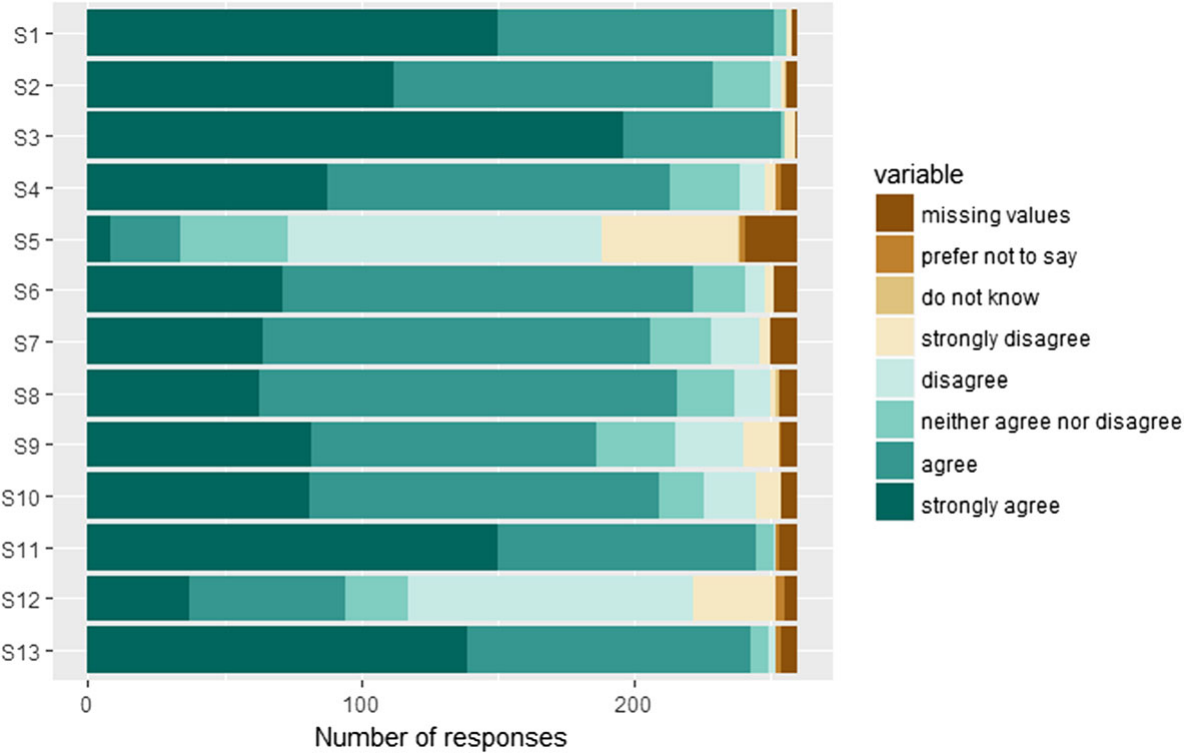
Frequency of responses to the 13 Likert-type statements (see Additional file 1: Appendix C for full statements S1-S13; see Additional file 4: Table S1 for means, counts, and percentages)

Means and frequencies of responses to the 13 Likert-type statements are presented in Fig. 1.

### Role of schools in early identification

On the questionnaire, 254 parents (98%) agreed or strongly agreed that it is important to identify MHD early in life (mean = 4.71, SD = 0.63), and 251 (97%) agreed or strongly agreed that schools have an important role in promoting pupils’ emotional health (mean = 4.54, SD = 0.62). The majority of parents (*N* = 229; 88%) agreed or strongly agreed that schools are well placed to detect emotional health difficulties (mean = 4.32, SD = 0.73).

### Benefits of screening

Most parents saw value in school-based screening. The majority of parents (*N* = 213; 82%) agreed or strongly agreed that screening would be helpful (mean = 4.15, SD = 0.89) but it is worth noting that some parents (*N* = 13; 5%) disagreed or strongly disagreed with this statement. A sizeable proportion of parents neither agreed nor disagreed that screening was helpful (*N* = 26; 10%).

Table 3 shows the themes pertaining to the benefits of screening, as included in any of the three open-ended responses. These responses corroborated the finding that parents valued screening. Parents’ comments on the benefits of screening can be broadly divided into benefits for children and benefits of the school setting in particular. In terms of benefits for children, many parents thought that screening would improve early identification and support of MHD, and that this early support could prevent future problems. On the whole, parents valued the role of schools in mental health provision, and indicated the usefulness of having an ‘outside’ perspective in addition to their own. Parents highlighted that schools could support pupils not only, but also parents and families. Furthermore, they believed that screening could have benefits for the schools themselves.

**Table 3:** Perceived benefits of MHD screening

Theme	Number of comments
*Theme 1. Benefits of screening for mental health difficulties for the child*	
Sub-theme 1. Screening can lead to early identification of MHD/(early) identification is important Ex. *“[E]arly detection helps in [getting] more help for the child”*	65
Sub-theme 2. Early identification of MHD can lead to early support Ex. *“I think they’d be a good idea, as some children will benefit from early intervention.”*	79
Sub-theme 3. Early identification of MHD can prevent future problems Ex. *“To spot the signs of any child that may be struggling. They would hopefully be offered guidance to avoid things like self-harming and suicide.”*	27
Sub-theme 4. Personal stories of experiences with MHD Ex. *“We have benefited already from our child receiving emotional support in school so think screening is a good idea.”*	18
Sub-theme 5. Screening is systematic/ involves *all* children Ex. *“Regular checks would make sure children do not ‘slip through the net’”*	9
*Theme 2. Benefits of the school setting*	
Sub-theme 6. School staff can provide valuable insight on mental health due to relationship to pupils Ex. *“The children spend a majority of their day at school, therefore you would assume the staff there know them quite well and could notice changes.”*	14
Sub-theme 7. School is a safe place for children and children trust their teachers Ex. “*The children often have a trusted relationship with their teachers and TAs and are in a safe space to explore their emotional wellbeing.”*	23
Sub-theme 8. Screening promotes an ‘all adult’ approach that involves parents, teachers, and staff in identification Ex. *“Every adult should be involved in ensuring kids are thriving”*	16
Sub-theme 9. School screening could benefit children who cannot get support at home/ who have problems at home Ex. *“May not have emotional support at home and best to talk to someone sooner rather than later, so that things don’t bottle up.”*	18
Sub-theme 10. Early identification could lead to improved academic performance/improved school life Ex. *“Early detection of emotional health problems. These problems can then be dealt with early so not to cause problems in their learning and overall wellbeing.”*	10
Sub-theme 11. Screening is also beneficial to schools themselves Ex. *“Surely it would benefit schools and there ciricculam [*sic*] if they knew the children who where struggling.”*	3
Sub-theme 12. Schools can support parents and families Ex. *“Could help parents of children with emotional health difficulties - offer support/advice”*	15

### Harms of screening

In total, 165 parents (63%) disagreed or strongly disagreed that screening would be harmful to children (mean = 2.33, SD = 1.13). Importantly, 34 parents (13%) agreed or strongly agreed that screening would be harmful, and 39 (15%) neither agreed nor disagreed.

Table 4 shows the themes relating to harms of screening, as included in any of the three open-ended responses. Comments can be divided into harms of MHD screening for the child, harms associated with screening accuracy/reliability, and harms associated specifically with identification in the school setting. Of the perceived harms to children, some were more minor and temporary than others, for example children feeling uncomfortable during screening or not understanding the process. More significant and lasting harms related to the singling out and potential stigmatisation of children and the lack of support available for identified pupils.

There were many concerns regarding the accuracy and reliability of screening. Parents were worried about the effects of false positive and false negative results. Some parents felt that it was difficult to identify MHD in children, and that schools could misunderstand children’s answers. There were also concerns about children giving false answers in order to please adults, or, more seriously, adults leading children into giving certain answers.

Finally, there were a number of concerns related to screening in the school setting. Several parents believed that schools should not use a whole-school approach to identification, and that identification should instead be done on a case-by-case basis by parents, mental health professionals, or individual teachers. There were further concerns that schools were already overwhelmed and could not add screening to their list of responsibilities. Finally, parents believed that school staff did not have proper training to identify MHD, and called for in-depth training before screening programme implementation.

**Table 4:** Perceived harms of MHD screening

Theme	Number of comments
*Theme 3. Harms of screening to the child*	
Sub-theme 13. Screening may be uncomfortable for children Ex. *“It could be harmful as an outsider could come in to discuss with the children and they could then worry as it’s an unfamiliar face and might not feel comfortable to talk.”*	11
Sub-theme 14. Children may feel singled out/ may be stigmatised Ex. *“Could make the child feel singled out if they were treated differently...”*	9
Sub-theme 15. Children may not understand the screening process or the questions Ex. *“Children wouldn’t know why they where taking part”*	4
Sub-theme 16. Once identified, there might not be resources to support children Ex*. “Obviously these checks would need to be done, recorded, and dealt with very sensitively and then are there resources to follow up any cases with concern? ...”*	6
*Theme 4. Harms associated with screening accuracy*	
Sub-theme 17. It is difficult to detect emotional difficulties in children Ex. *“How often screening? Kids are up one week, down the next, in fact emotions change daily!”*	16
Sub-theme 18. Schools may misunderstand or misinterpret children’s answers to screening questions Ex. *“But I do feel that it may not seem clear to school, why these problems could be present, which could cause problems, so understanding is needed, especially with special needs.”*	13
Sub-theme 19. Teachers or staff might ask leading questions Ex. *“The health checks should not be giving children any ideas about how they ‘should’ feel and so long as they are giving an unbiased assessment, should not be harmful.”*	6
Sub-theme 20. Children may try to say the ‘right’ thing to adults/ tell them what they want to hear Ex. *“Children are often keen to please adults and may feel the need to answer what they think people want to hear.”*	10
Sub-theme 21. Screening could result in false positive or false negative results Ex. *“Dependent on the reliability and validity of the screening tool. A little wary of over-reliance on a tool.”*	20
*Theme 5. Harms associated with screening at school*	
Sub-theme 22. Mental health begins at home/ is the responsibility of parents Ex. *“I do not believe its [sic] the schools responsibility to identify emotional health difficulties. It should be a parental responsibility between them and health professionals.”*	8
Sub-theme 23. Identification of MHD should be done by mental health professionals Ex. *“This worries me because I think this is an area that should be dealt with by mental health specialists...”*	5
Sub-theme 24. Screening could be harmful when conducted without proper training Ex. *“Staff conducting the checks would require suitable training/support. Poorly trained staff may mis-read emotions and information given by children.”*	13
Sub-theme 25. Schools are already overwhelmed/screening would overwhelm schools Ex. *“Within a strong framework. Good schools could probably cope but struggling schools may be overwhelmed by any additional requirements.”*	8
Sub-theme 26. Screening is unnecessary because teachers should be able to recognize MHD Ex. *“Deemed to be part of teachers training to spot signs.”*	5
*Theme 6. No harms*	
Sub-theme 27. Screening is not harmful (explicitly stated) Ex. *“Never harmful to develop a child’s emotional development.”*	22

### Implementation of screening programmes

Most parents (*N* = 222; 85%) agreed or strongly agreed that they would be prepared to fill out questionnaires on their child’s emotional health. Approximately as many agreed or strongly agreed that they would be prepared for teachers (*N* = 216; 83%) or their children (*N* = 206; 79%) to fill out questionnaires. About 8% of parents did not have a strong opinion regarding who should fill out emotional health screening measures. There was no clear consensus regarding the way in which parental consent should be sought; 186 parents (72%) endorsed opt-in consent and 209 (80%) endorsed opt-out, signifying that some parents endorsed both options.

The vast majority (*N* = 245; 94%) of respondents agreed or strongly agreed that parents should receive individualised feedback on their child’s emotional health, but they were divided on whether feedback should be distributed to all parents, or just to those parents whose children were indicated to be at-risk by the screening programme. While approximately half of respondents (*N* = 135; 52%) believed that parents should receive feedback regardless of screening results, 94 parents (37%) indicated that parents should receive feedback only if their child was identified as at-risk by the screening. All but 11 respondents (less than 5%) agreed or strongly agreed that they would be willing to work with schools or with other organisations to ensure that their child received any necessary support.

Fisher’s exact tests did not indicate any significant differences in responses by gender, ethnicity, or school for any of the thirteen statements (*p* > 0.05 for all statements). Age and statement responses were weakly correlated (polyserial correlations ranged from − 0.06 to 0.21.)

## Discussion

This study examined UK parents’ views on the conceptual acceptability of school-based screening for MHD in primary schools. Overall, parents endorsed the importance of early identification and viewed the school as an appropriate setting for screening. Parents generally believed that screening would be helpful, and valued screening’s potential to identify difficulties, indicate need for support, and prevent future problems. Parents further valued teachers’ insight into pupils’ mental health and schools’ ability to support parents. However, one in eight parents who participated in the study believed that MHD screening programmes could be harmful, citing inaccurate identification, stigmatisation, and low availability of follow-up care as key harms. There was no clear consensus on desirable components of screening programmes. Parents did not agree on how they would prefer to give consent (some parents endorsed both opt-in and opt-out options), and while nearly all parents believed feedback should be given, they were divided on whether it should be provided only for those experiencing MHD. Most parents were happy for children or teachers to complete screening questionnaires, or to complete them themselves.

Parents’ general support for screening is in keeping with other studies on school-based identification [50–55]. Two other UK-based studies on specific screening programmes also reported that parents found screening acceptable [54, 55], although one programme required modifications to enhance acceptability [55]. A similar US-based study of a primary school screening programme for social and emotional problems also reported that 93% of parents found the screening appropriate [53]. Furthermore, parents’ comments on screening’s potential to identify problems, support children and parents, and offer a safe space for pupils have also been cited in other studies from Australia and the United States [52, 53, 56–58].

Parents’ concerns about the accuracy of results represent a key consideration in the implementation of MHD screening programmes, and are also reflected in other health screening programmes (e.g. those for breast cancer [59] or prostate cancer [60]). Indeed, empirical evidence indicates high rates of false positive results for school-based MHD, for example, 20% for social anxiety disorder screening [61] and 43% for suicide screening [62]. The possibility of both false negative and false positive results must be addressed explicitly and carefully to avoid reducing referrals among children with false negative results and anxiety among those with false positive results.

Parents’ worries about potential stigmatisation reflect those of a similar study of American parents’ attitudes toward depression and suicide screening [50], which found that 60% of parents were concerned about labelling students and 43% believed identified students would be treated unfairly. Stigma and discrimination are significant concerns, as they can exacerbate MHD [63] and dissuade CYP from accessing care and support [64]. While stigma is a key barrier to programme acceptability [24, 27, 37], the potential for harm can be limited through mental health literacy training for educators [65] and sensitive handling of the identification and feedback process [23].

Finally, parents’ concerns about availability of follow-up care are also reflected in the literature. For example, in Nadeem and colleagues’ US-based study of a school-based identification programme for suicide risk, many of the involved parents did not seek mental health resources for their children due to distance, lack of time, or long waiting lists [58]. Lack of resources for follow-up care is a particular concern in the UK, where there is a significant unmet need for mental health care [66, 67]. While the government’s plan to improve school-based mental health care and support has potential to reduce this treatment gap [32], the success of these measures will be dependent on the availability of enough early intervention and specialist services to meet identified need.

Parents’ views on key programme components (e.g. consent, feedback) provide practical input into intervention design. Obtaining parental consent is a challenge for MHD identification programmes [68]. While active (opt-in) consent is highly recommended, it requires significant human and time resources [23]. Passive (opt-out) consent generally yields greater participation in screening, especially among those at high risk for MHD [69]. Parents’ overwhelming support for feedback is interesting given previous findings from US-based studies that not all parents are receptive to feedback when offered [68, 70]. However, our results report on theoretical acceptability, whereas the other two studies examined feedback as part of an intervention.

The lack of variation in response by parent gender, ethnicity, or age is consistent with previous acceptability studies. A similar US study of parent acceptability of depression and suicide screening found that parents’ age and gender did not affect their views on programme acceptability. And, although White parents were significantly more likely to support suicide screening compared to non-White parents, this relationship did not hold for depression screening [50]. However, given the relative homogeneity of our sample, it is also possible that our study was underpowered to find differences across subgroups.

### Strengths and limitations

This study offers new and valuable information in terms of the public mental health response to MHD in CYP. Our focus on primary school aged children is important given the relative lack of school-based identification programmes for this age group as compared to older students [38]. The survey response rate of 61% is higher than those of similar surveys, which all attained response rates of less than 50% [50, 71–73]. Furthermore, the inclusion of open-ended comments allowed parents to explain their views on screening and its potential harms and benefits. The survey was also one of the first to offer UK-based evidence on the acceptability of screening, which is important given that beliefs about MHD identification may vary by context and culture [24].

We also acknowledge several limitations. First, since there was no existing measure of attitudes about MHD screening, we developed a new measure, which may limit the generalisability of results. Second, following PPI and stakeholder feedback, the questionnaire was simplified and key questions about family life and educational qualifications were excluded. This information would have been useful for understanding whether results differed across groups of parents. Third, anonymous participation was incentivised through use of a prize draw, which might have encouraged duplicate entries. Fourth, we only focused on parents of primary school children. Finally, our sample size was relatively small, and our respondents were largely homogenous in terms of key socio-demographic characteristics, which limits the generalisability of our results. Responding parents were from four relatively deprived areas in two UK counties, 95% of respondents were of White British ethnicity, and 80% were mothers or female guardians. The lack of diversity in our sample is a significant limitation because parents of underrepresented groups may hold different views on the acceptability of screening. Indeed, there is wide cultural variation in views on mental health, and any screening programme must be considerate of these differences [24].

### Implications for practice

While school-based screening for MHD has the potential to improve psychosocial outcomes for CYP as part of a multi-tiered system of mental health identification and support, it is first necessary to establish evidence of its effectiveness, acceptability, and feasibility. Determining parents’ views on school-based screening represents an important first step for understanding whether screening programmes for MHD could be feasible and sustainable. Currently, 99% of UK schools report taking action to identify pupils with MHD, but most schools rely on ad-hoc identification by school staff [12]. Schools that do systematically screen for MHD often do so with non-validated measures [12], and many currently-implemented school-based mental health programmes are not evidence-based [11]. In providing evidence of parents’ support for systematic identification, this study supports the further development of primary school screening programmes for MHD.

However, acceptability is just one aspect of the screening guidelines as given by the National Screening Committee. In addition to being acceptable, a screening programme should also be effective in reducing morbidity/mortality, balanced in terms of harms and benefits, and economically feasible [40]. It is currently unclear whether school-based MHD screening would fulfil these other criteria, as evidence on the effectiveness, cost effectiveness, and feasibility of screening is mixed [38, 74]. The potential for harm caused by screening must also be further explored: a recent review of school-based screening across all types of MHD [74] found only two studies in the literature that assessed iatrogenic effects of school-based MHD screening (neither of which found an iatrogenic effect) [52, 75]. Furthermore, the National Screening Committee highlights that there should be accessibility to an effective intervention for any identified individuals [40]. Given that many CYP have limited access to mental health care [21], further investigation is needed into this criterion before the wider implementation of school-based MHD screening programmes.

### Implications for future research

More evidence on the acceptability of school-based MHD identification programmes is needed before their widespread implementation in the UK. In particular, future studies must seek to understand the acceptability of screening for parents of minority ethnic groups as well as fathers and male guardians, as these views were under-represented in the current study. Also, as this survey only targeted parents, further research is needed to establish acceptability amongst school staff and mental health professionals. Additionally, the survey focused on identification in primary schools and may not generalisable to other age groups, suggesting a need for more research on programmes designed for older pupils. Finally, this study only reported on school-wide screening for mental health difficulties. Researchers should also seek to understand the acceptability of 1) other types of identification models (e.g. curriculum-based or staff training/teacher nomination models), and 2) more targeted identification of specific types of mental health problems (e.g. risk of suicide).

## Conclusions

This survey provides evidence that, in the socio-demographic context of our study, most parents will accept mental health difficulties screening within primary schools, and that school-based screening is a viable model for identification. The findings on potential harms as well as suggestions for programme implementation are valuable to the on-going development and evaluation of acceptable and sustainable school-based identification models. Implementation and scale-up of such programmes will require further understanding of the perspectives of mental health professionals, children, school staff, and the general public.

## Additional files

Additional file 1: Appendix C. Means and frequencies of responses to Likert-type items. Appendix C. Formatted parent questionnaire. (DOCX 290 kb)

Additional file 2: Appendix A. Parent invite letter. (DOCX 568 kb)

Additional file 3: Appendix B. Participant information sheet. (DOCX 532 kb)

Additional file 4: **Table S1**. Means and frequencies of responses to Likert-type items. (XLSX 10 kb)

## Abbreviations

CYP: Children and young people
MHD: Mental health difficulties
NHS: National Health Service
PPI: Patient and public involvement
UK: United Kingdom
US: United States
