# Supplementary material for: Acceptability of screening for mental health difficulties in primary schools: a survey of UK parents
Source: BMC Public Health. 2018 Dec 22;18:1404. doi: 10.1186/s12889-018-6279-7 (PMC6303970; doi:10.1186/s12889-018-6279-7)
Supplement: Supplementary file 1 — Appendix C. Means and frequencies of responses to Likert-type items. Appendix C. Formatted parent questionnaire. (DOCX 290 kb) [file 12889_2018_6279_MOESM1_ESM.docx]

**Appendix C. Formatted parent questionnaire**

**
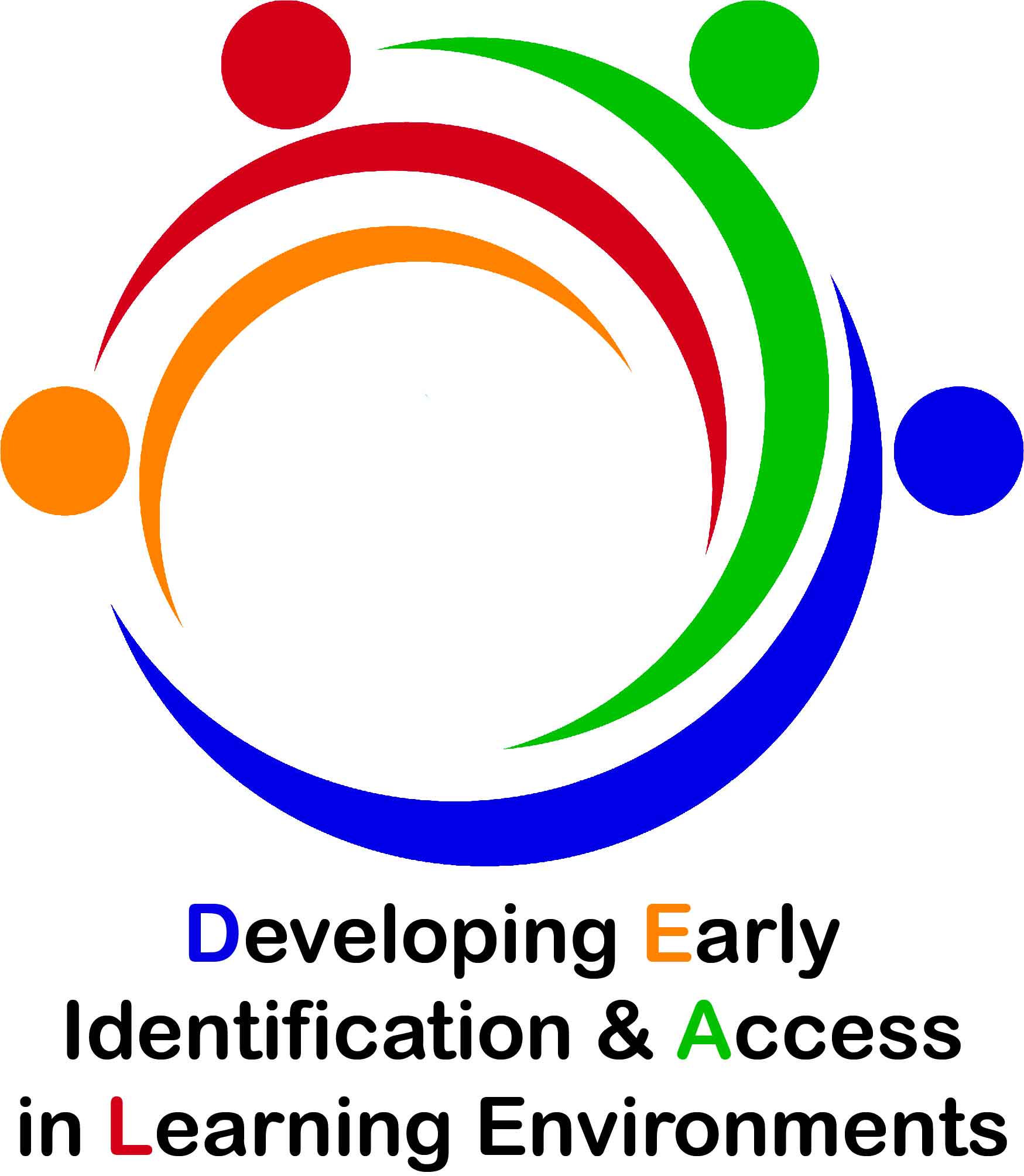
**

**What is this questionnaire for?**

- *This questionnaire asks parents and caregivers if it would be a good idea for their children’s school to perform routine checks or* ***screening*** *of children’s emotional health.*
- Screening is just one way that schools may be able to improve the way they identify children, but this would only be the case if parents think it could be helpful.

**What do you mean by emotional health and emotional health difficulties?**

- Emotional health is about how we think, feel and behave. Good emotional health is about feeling positive about daily life and work or school and having good relationships with family and friends. When a person is experiencing emotional health difficulties they may have been feeling down, stressed, worried or angry for a period of time, or they may be experiencing problems in getting on with, or relating to family and friends.

**What do you mean by ‘routine checks’ or screening?**

- By routine checks or screening of children’s emotional health, we mean the collection of information about the emotional health and wellbeing of all children in a school.
- The purpose of screening is to detect people with the early signs of a problem so that they can be offered help at the earliest opportunity.
- Schools already use a similar approach to monitor children’s physical health by measuring height and weight in children’s first and last years of primary school. This information is used to provide advice to parents about how to improve their child’s physical health.

**How should I complete this questionnaire?**

- Please try and answer all of the questions. There are **no right or wrong answers**; we are interested in your opinions about this issue.
- It should take you about 10 minutes to complete the questionnaire
- All of the questions have a ‘don’t know’ or ‘prefer not to say option’ and it is better to use one of these options rather than leaving a question blank. There are also free text boxes at the end of the questionnaire; please use these boxes to tell us any additional thoughts you have.

**Your views about routine emotional health checks or screening in schools**

Please rate your agreement with the following statements. Tick the box on the scale that best describes how much you agree or disagree with the statement. Tick ***one*** box only for each question.

1. Schools have an important role in making sure that children have good emotional health

**Strongly Disagree Neither agree Agree Strongly Don’t know Prefer not to say**

**disagree nor disagree agree**

☐ ☐ ☐ ☐ ☐ ☐ ☐

1. Schools are well placed to spot the early signs that a child may be experiencing emotional health difficulties

**Strongly Disagree Neither agree Agree Strongly Don’t know Prefer not to say**

**disagree nor disagree agree**

☐ ☐ ☐ ☐ ☐ ☐ ☐

1. It is important to identify children experiencing emotional health problems as early as possible

**Strongly Disagree Neither agree Agree Strongly Don’t know Prefer not to say**

**disagree nor disagree agree**

☐ ☐ ☐ ☐ ☐ ☐ ☐

1. It would be *helpful* if schools carried out routine emotional health checks (screening) on their pupils to assist them in identifying children who may be experiencing emotional health difficulties. Please give reasons below.

**Strongly Disagree Neither agree Agree Strongly Don’t know Prefer not to say**

**disagree nor disagree agree**

☐ ☐ ☐ ☐ ☐ ☐ ☐

Comments:

1. It would be *harmful* if schools carried out routine emotional health checks on their pupils. Please give reasons below.

**Strongly Disagree Neither agree Agree Strongly Don’t know Prefer not to say**

**disagree nor disagree agree**

☐ ☐ ☐ ☐ ☐ ☐ ☐

Comments:

1. I would be prepared to complete a questionnaire about my child's emotional health for the purpose of a routine emotional health check (screening).

**Strongly Disagree Neither agree Agree Strongly Don’t know Prefer not to say**

**disagree nor disagree agree**

☐ ☐ ☐ ☐ ☐ ☐ ☐

1. I would be prepared for my child to complete a questionnaire about his/her own emotional health during school time for the purpose of a routine emotional health check (screening)

**Strongly Disagree Neither agree Agree Strongly Don’t know Prefer not to say**

**disagree nor disagree agree**

☐ ☐ ☐ ☐ ☐ ☐ ☐

1. I would be prepared for a teacher to complete a questionnaire about my child’s emotional health for the purpose of a routine emotional health check (screening)

**Strongly Disagree Neither agree Agree Strongly Don’t know Prefer not to say**

**disagree nor disagree agree**

☐ ☐ ☐ ☐ ☐ ☐ ☐

1. Schools should ask for parents’ written permission each time that routine emotional health checks are carried out (opt in consent)

**Strongly Disagree Neither agree Agree Strongly Don’t know Prefer not to say**

**disagree nor disagree agree**

☐ ☐ ☐ ☐ ☐ ☐ ☐

1. Schools should tell parents when emotional health checks are taking place. Parents can then inform the school if they do not wish for their child to be included in the checks (opt out consent)

**Strongly Disagree Neither agree Agree Strongly Don’t know Prefer not to say**

**disagree nor disagree agree**

☐ ☐ ☐ ☐ ☐ ☐ ☐

1. Parents should be provided with feedback about their child’s emotional health following his/her participation in an emotional health check

**Strongly Disagree Neither agree Agree Strongly Don’t know Prefer not to say**

**disagree nor disagree agree**

☐ ☐ ☐ ☐ ☐ ☐ ☐

1. Parents should receive feedback only if their child is identified as experiencing emotional health difficulties

**Strongly Disagree Neither agree Agree Strongly Don’t know Prefer not to say**

**disagree nor disagree agree**

☐ ☐ ☐ ☐ ☐ ☐ ☐

1. I would be happy to work with the schools and other organisations such as the NHS, if an emotional health check showed that my child could benefit from extra support

**Strongly Disagree Neither agree Agree Strongly Don’t know Prefer not to say**

**disagree nor disagree agree**

☐ ☐ ☐ ☐ ☐ ☐ ☐

Please share any thoughts or comments that you may have in relation to emotional health checks in schools, or the DEAL study more generally.

**A bit about you**

We need to gather this information to understand whether the people responding to this questionnaire are reflective of all parents and caregivers in the school, and whether we have a good spread of views across parents and caregivers of children of different ages.

1. Please write the number of children you have in each school year

Reception: ____ Year 1: ____ Year 2: ____ Year 3: ____

Year 4: ____ Year 5: ____ Year 6: ____

1. What is your gender?

☐ Male ☐ Female ☐ Transgender ☐ Other ☐ Prefer not to say

1. How old are you? Please write your age in the box
2. What is your ethnic group? Choose one option that best describes your ethnic group or background

White Mixed / Multiple ethnic groups

☐ English / Welsh / Scottish / ☐ White and Black Caribbean

Northern Irish / British ☐ White and Black African

☐ Irish ☐ White and Asian

☐ Gypsy or Irish Traveller ☐ Any other Mixed / Multiple ethnic

☐ Any other White background, please describe: background, please describe:

Asian / Asian British Black / African / Caribbean / Black British

☐ Indian ☐ African

☐ Pakistani ☐ Caribbean

☐ Bangladeshi ☐ Any other Black / African / Caribbean

☐ Chinese background, please describe:

☐ Any other Asian background,

please describe:

Other ethnic group ☐ Prefer not to say

☐ Arab
☐ Any other ethnic group, please describe

………………………………………………………………………………Detach slip and return separately…….......

1. ***Please tick the box*** and provide your name and contact details if you would be prepared to take part in an interview with a researcher from the University of Cambridge to talk a bit more about your answers.

NAME_____________________________________

EMAIL_____________________________________ Telephone__________________________
