# Supplementary material for: Acceptability of screening for mental health difficulties in primary schools: a survey of UK parents
Source: BMC Public Health. 2018 Dec 22;18:1404. doi: 10.1186/s12889-018-6279-7 (PMC6303970; doi:10.1186/s12889-018-6279-7)

**Appendix A. Parent invite letter**

| CLAHRC East Of England  Douglas House  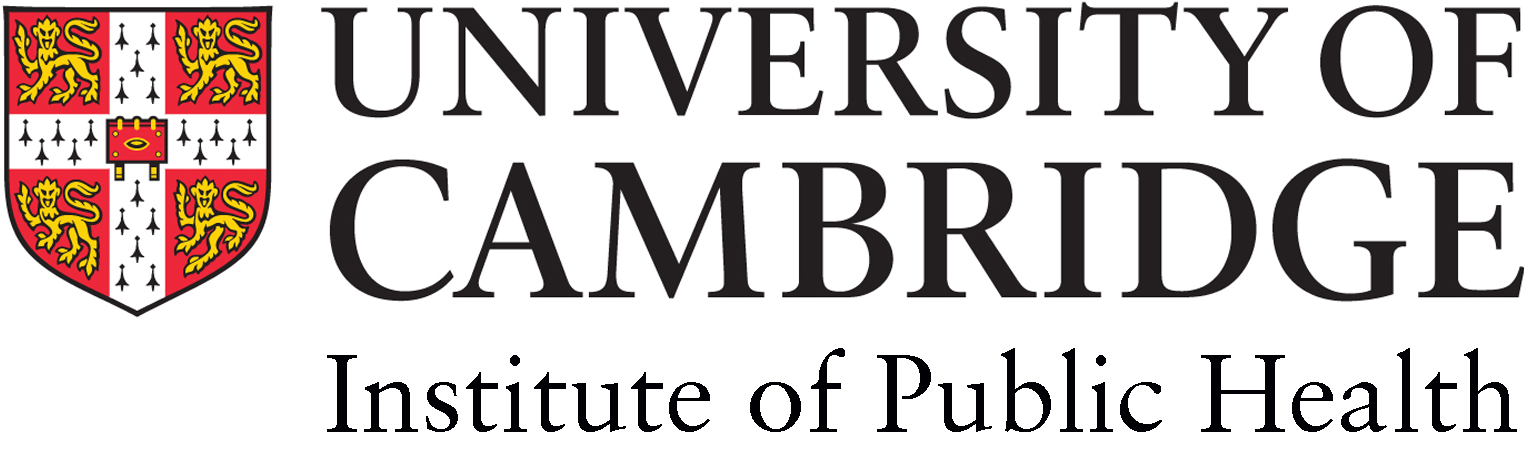18 Trumpington Road  Cambridge CB2 8AH Tel: 01223 465194 Email: [emma.howarth@medschl.ac.uk](mailto:emma.howarth@medschl.ac.uk)  Website: clahrceoe.nihr.ac.uk |
| --- |
|  |

July 2017

Dear parent or caregiver,

RE: **Invitation to participate in the ‘DEAL’ Study – Improving children’s wellbeing**

My name is Dr Emma Howarth, and I am writing to invite you to participate in a research study. The study aims to find the best ways for schools to spot early signs of children’s emotional health difficulties, and is called ‘DEAL’, which stands for ‘Developing Early identification and Access in Learning Environments’. We hope to contribute important information about the best way for schools to support children and families to live happy and healthy lives. More detailed information about the study is included in the information sheet enclosed with this letter.

**Participation in this study will involve you filling out a short questionnaire that should take no longer than 10 minutes to complete.** This questionnaire can be completed online via a link sent to you by the school, or on paper which can be returned to the school using the freepost envelope. You only need to complete the questionnaire once, even if you have more than one child at the school.

To make this study a success we need as many parents as possible from the school to complete a questionnaire. Each questionnaire returned to the school will be **entered into a prize draw with the chance to win £50 of shopping vouchers**. You may also wish to tick the option to participate in an interview, where you would be invited to talk to me, or another researcher, about your thoughts regarding the role of schools in supporting children’s emotional health and wellbeing.

We very much hope that you feel able to participate in this study, and please feel free to contact me with any questions or queries.

Yours faithfully,


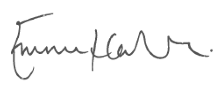


Dr Emma Howarth, Senior Research Associate


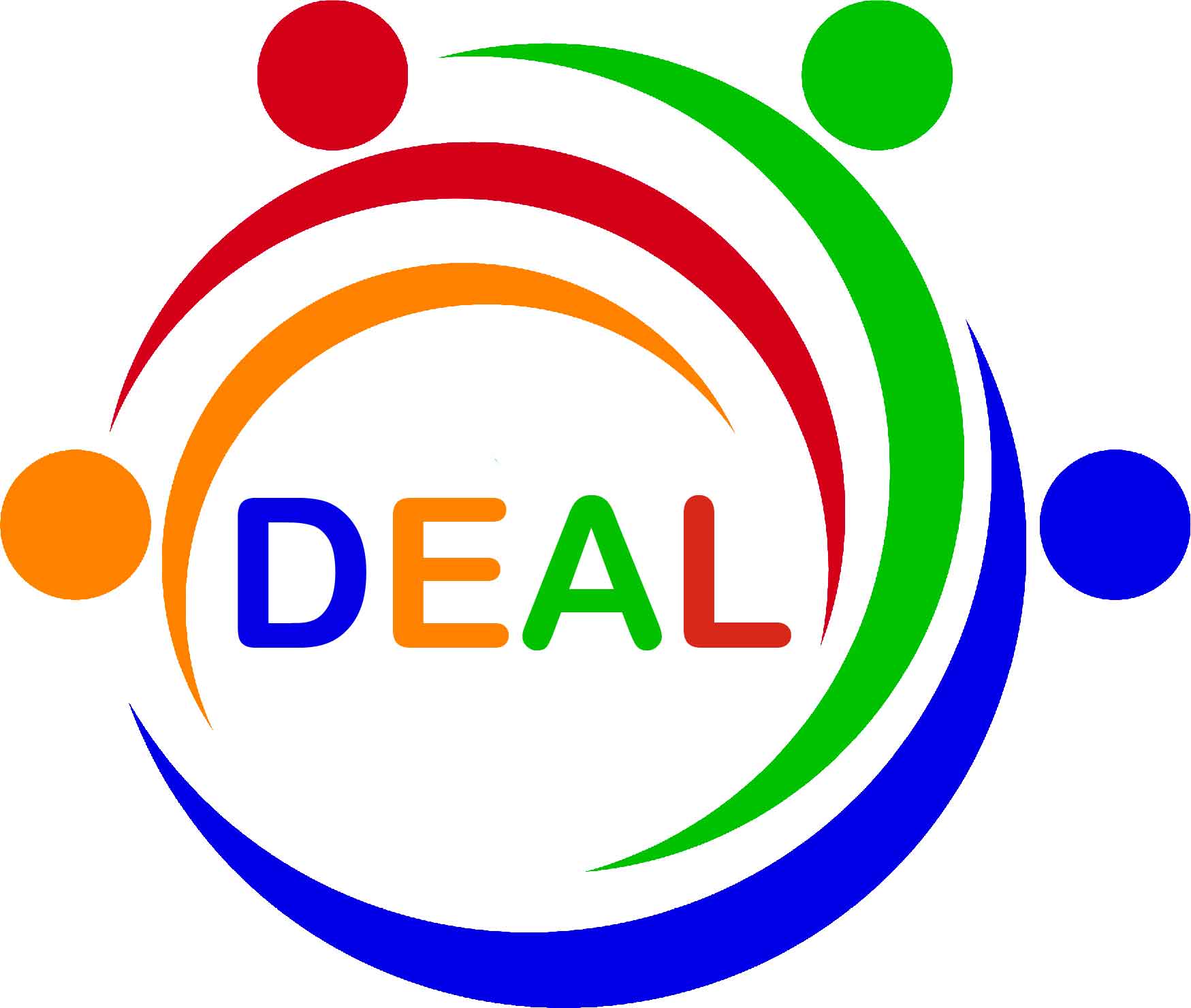

Supplement: Supplementary file 2 — Appendix A. Parent invite letter. (DOCX 568 kb) [file 12889_2018_6279_MOESM2_ESM.docx]
