# Supplementary material for: Acceptability of screening for mental health difficulties in primary schools: a survey of UK parents
Source: BMC Public Health. 2018 Dec 22;18:1404. doi: 10.1186/s12889-018-6279-7 (PMC6303970; doi:10.1186/s12889-018-6279-7)
Supplement: Supplementary file 3 — Appendix B. Participant information sheet. (DOCX 532 kb) [file 12889_2018_6279_MOESM3_ESM.docx]

**Appendix B. Participant information sheet**


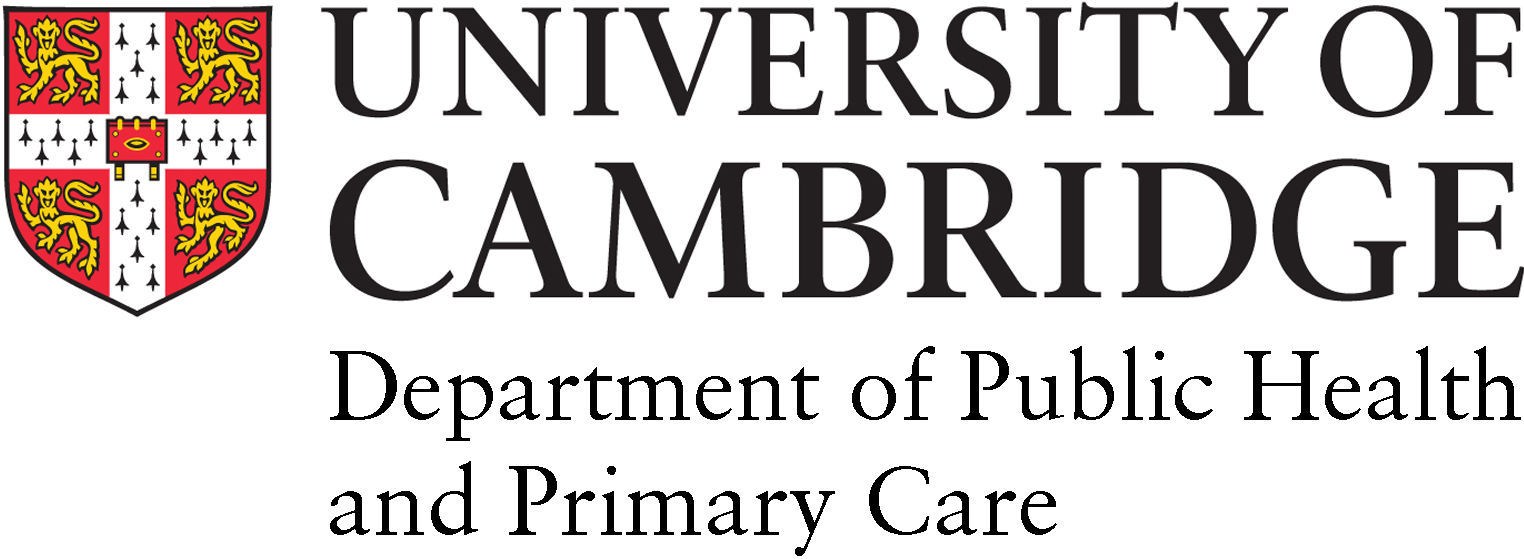

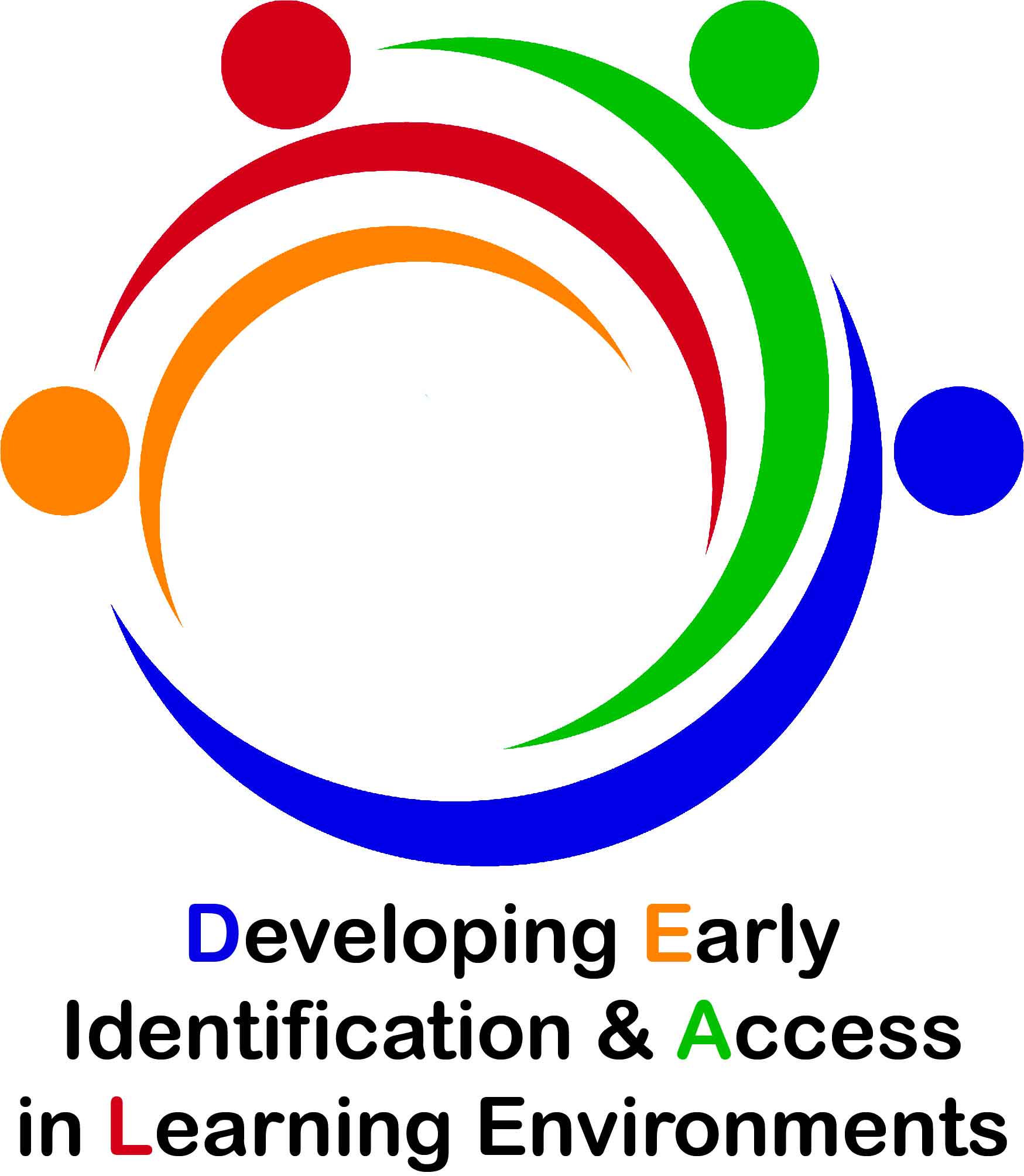


**The ‘DEAL’ STUDY**

**Developing Early identification and Access in Learning environments**

**Participant Information Sheet**

We are a team of researchers working at the University of Cambridge, and we are inviting you to take part in a study to find out about the best ways for schools to spot early signs of children’s emotional health difficulties. This study is called the ‘DEAL’ study, which stands for ‘Developing Early identification and Access in Learning Environments’.

*Before you decide whether or not to take part in this study it is important for you to understand why the research is being done and what it will involve. Please take time to read the following information carefully and discuss it with others if you wish. You are welcome to contact a member of the research team (please see details below) if there is anything that is not clear or if you would like more information. You can also contact the head teacher or member of school staff listed at the end of the sheet.*

**What is the purpose of the study?**

- It is estimated that in the UK one in ten children aged 5-16 experience some emotional health or behavioural difficulty and would benefit from having extra support. Research tells us that support is much more effective if it is offered at the first sign of a problem, rather than later when problems have got worse.
- Schools are well placed to spot children who would benefit from extra support, however, schools teachers and other staff are not always equipped to recognise the early warning signs that a child may be experiencing emotional difficulties. This can sometimes mean that children do not get the right help at the right time, which might mean that problems get worse.
- Through the DEAL study we want to find out how best to support schools to spot the early warning signs of emotional difficulties so that children can get the right help as soon as possible, enabling children to lead happy and healthy lives and achieve their full potential.

**Why have I been asked to take part in this study?**

- We are asking **all** parents with a child attending the school to fill out a short questionnaire to tell us what they think. This will be anonymous, meaning that we will not know which parents have responded.
- We will only contact those parents who say that they might be interested in taking part in an interview and provide us with their contact details.

**What would I have to do?**

Completing the questionnaire

- You would be asked to fill out a short questionnaire. There will be a number of options for completing the questionnaire – online or by post.
- Each questionnaire returned to the school will be entered into a prize draw with a chance of winning £50 of shopping vouchers. There will be a raffle ticket on questionnaires or numbers generated by online completion which you should keep safe so that we can identify winners.
- If you do not want to fill out the questionnaire but would like to be entered into the draw, please take off the raffle ticket and send back a blank questionnaire. If you are completing an electronic version please tick the box to indicate that you would like to be entered into the prize draw. Please take a screen shot of your lottery number or print it out.
- On the questionnaire there is an option to say if you would be happy to take part in an interview (of up to an hour) with a researcher. You will be asked to complete the permission form and return it to the school with your questionnaire. A researcher may then contact you to set up a time for the interview. We may not contact everybody who is interested in taking part in this part of the study.

Taking part in an interview

- If you are happy to be contacted about taking part in an interview, please tick the relevant box on the questionnaire and provide us with your contact details.
- An interviewer may contact you to arrange a time to meet with you at the school, or in another location that is convenient for you to explain the purpose of the interview in more detail. If you agree to be interviewed the researcher will ask you to provide your written consent before going ahead.
- We will ask your permission to tape record the interview and also take some notes. These tapes will be destroyed at the end of the study. If you do not wish for your interview to be taped it will still be possible for you to take part.
- You will be asked a series of questions about your experiences of having a child in primary school and your opinion on what you think would be a good way for schools to identify children who may need additional support.
- You do not need to be an expert or have any experience of research, teaching or other professional fields. We are interested in your thoughts, in your own words.
- You will receive a £10 shopping voucher as a token of appreciation of your time.

**Why should you take part?**

- Very little is known about how best to identify children in school settings who might need extra support.
- We are interested to find out what schools are already doing to identify children who may need extra support, and to hear the views of parents, school staff and local mental health workers about the most acceptable and practical ways to do this. As a parent of a child attending primary school you will have a unique view on how your child’s needs are addressed by their school, what could be changed or improved, and what you think would be best for your child. Your opinion is very important as it will help us develop a programme that is acceptable and useful for families and schools.
- You wouldn’t be expected to provide solutions, but to tell us about your experiences and thoughts, in your own words.

**What are the advantages to taking part?**

- There will not be any direct benefits to you or your child, although taking part in this study will help us to understand the best ways for schools to identify the early warning signs that children are experiencing emotional difficulties and to ensure that parents’ ideas inform the development of an early identification system.

**Are there possible disadvantages and/or risks in taking part?**

- Participating in this study will take between 20 minutes (questionnaire only) to 80 minutes (questionnaire + interview).
- If you decide to take part in an interview, talking about negative experiences (if there are any) could make you feel uncomfortable. If this happens, you may ask the researcher to skip questions that touch on topics you would rather not talk about, or stop the interview all together.
- The researcher will also be able to advise you of other people or services to talk to following the interview, should you want to.

**Will my participation in this study be kept confidential?**

- Your participation in the research will be kept strictly confidential. Completing a questionnaire will not require you to give any identifying information. This means your responses can not be linked with your name.
- You can choose to meet with a researcher on school premises or another location that is convenient for you.
- If you take part in an interview we will make sure that we remove any identifying information when the interviews are written up (transcribed).
- The audiotapes of the interviews will be securely stored at the university, with personal details kept in a locked file or secure computer with access only by the immediate research team.
- Please note that the only exception to this confidentiality will be if you disclose information which suggests a risk of serious danger to any person (including yourself).
- At that point we will share information for safety reasons, but where possible we will try to let you know first.

**What will happen to the results of the research study?**

At the end of the study we will send you a summary describing what we found. We also plan to publish the results and present the findings to each school, as well as regional and local decision makers. There will be no mention of your name or any other personal details that could identify you.

**Do I have to take part?**

It is up to you to decide whether or not you will take part in the study. If you do decide to take part you are still free to withdraw at any time and without giving a reason. A decision to withdraw at any time, or a decision not to take part, will not be communicated to the school and will not affect the treatment that your child receives from the school.

**Who can I talk to if I have any questions?**

The research team, head and school staff will be really pleased to answer any questions you have.
***Contact for further information***
Dr Emma Howarth, Principal investigator, University of Cambridge
[emma.howarth@medschl.cam.ac.uk](mailto:emma.howarth@medschl.cam.ac.uk)
Dr Jasmine Childs-Fegredo, Research Associate, University of Cambridge
[jc2039@medschl.cam.ac.uk](mailto:jc2039@medschl.cam.ac.uk)
HEAD: Name Link person: Name

***This research project has received ethical approval from the University of Cambridge Psychology Research Ethics Committee PRE.2017.045, and funded by the National Institute of Health Research***
